# Combined Administration of Fibrinogen and Factor XIII Concentrate Does Not Improve Dilutional Coagulopathy Superiorly Than Sole Fibrinogen Therapy: Results of an In-Vitro Thrombelastographic Study

**DOI:** 10.3390/jcm10102068

**Published:** 2021-05-12

**Authors:** Emmanuel Schneck, Marcus Muelich, Melanie Markmann, Fabian Edinger, Nina Cooper, Annette Moeller, Gregor Bein, Andreas Hecker, Christian Koch, Michael Sander, Matthias Wolff

**Affiliations:** 1 Department of Anesthesiology, Operative Intensive Care Medicine and Pain Therapy, University Hospital of Giessen, 35392 Giessen, Germany; m.muelich@web.de (M.M.); melanie.markmann@chiru.med.uni-giessen.de (M.M.); fabian.edinger@chiru.med.uni-giessen.de (F.E.); christian.koch@chiru.med.uni-giessen.de (C.K.); michael.sander@chiru.med.uni-giessen.de (M.S.); Matthias.wolff@chiru.med.uni-giessen.de (M.W.); 2 Institute for Clinical Immunology and Transfusion Medicine, Justus Liebig University, 35392 Giessen, Germany; Nina.Cooper@immunologie.med.uni-giessen.de (N.C.); Anette.Bohnert@immunologie.med.uni-giessen.de (A.M.); Gregor.Bein@immunologie.med.uni-giessen.de (G.B.); 3 Department of General & Thoracic Surgery, University Hospital of Giessen, 35392 Giessen, Germany; Andreas.hecker@chiru.med.uni-giessen.de

**Keywords:** hemorrhage, major bleeding, coagulation, thrombelastometry, impedance aggregometry, trauma

## Abstract

The early administration of fibrinogen has gained wide acceptance for the treatment of major hemorrhage, whereas the substitution of coagulation factor XIII (FXIII) is only supported by a low level of evidence. This study aimed to answer the question of whether a combined therapy of fibrinogen/FXIII substitution performs superiorly to sole fibrinogen administration in the treatment of dilutional coagulopathy. An in-vitro model of massive transfusion was used to compare the effect of combined fibrinogen/FXIII administration to that of sole fibrinogen therapy for the treatment of dilutional coagulopathy. For this purpose, the blood of red blood cell concentrates, fresh frozen plasma, and platelet concentrates were reconstituted in a ratio of 4:4:1, and then diluted with gelatin by 20% and 40%, respectively. Clot formation and stability were analyzed by thrombelastography. Both sole fibrinogen therapy (equivalent to 50 mg/kg) and the combined administration of fibrinogen (equivalent to 50 mg/kg) and FXIII (equivalent to 75 International Units (IU)/kg) increased fibrinogen-dependent mean clot firmness independently of the degree of dilution (20% dilution: 7 (6.3–7.8) mm; 20% dilution fibrinogen: 13.5 (13–17.3) mm; 20% dilution fibrinogen/FXIII: 16.5 (15.3–18.8) mm; 40% dilution: 3 (2–3.8) mm; 40% dilution fibrinogen: 8 (7–11.3) mm; 40% dilution fibrinogen/FXIII: 10 (8.3–11.8) mm; all *p* < 0.01). However, no differences were identified between the two treatment arms. Compared to fibrinogen therapy, no beneficial effect of the combined administration of fibrinogen and FXIII for the treatment of dilutional coagulopathy was detected in this in-vitro massive transfusion model. The result was independent of the degree of dilution.

## 1. Introduction

Severe hemorrhage following major trauma represents a common cause of death and continues to be associated with high mortality [1,2]. The main driver of death remains the development of coagulopathy, which is detectable in approximately 30% of all severe trauma patients. Dilutional coagulopathy induced by liberal fluid resuscitation is especially associated with adverse outcomes [3,4,5]. Even though the treatment of trauma-induced coagulopathy (TIC) can be started at the site of the incident, the sole presence of TIC correlates with an increase in mortality [1,6,7,8,9]. This is caused by the complex pathophysiological reactions that are connected to TIC [9,10]. First, trauma-induced shock leads to hypovolemia, hypoperfusion, and acidosis. Second, endothelial injury causes the release of tissue factor, leading to a procoagulatory burst via the generation of thrombin–thrombomodulin complex and activation of platelets [1]. Since fibrinogen stabilizes the clot by supporting platelet aggregation and fibrin polymerization, the trauma-induced decrease of fibrinogen (normal value 2.0–4.0 g/L) plays a crucial role in the development of TIC [10,11]. Last, the disbalance of increased levels of tissue plasminogen activator (t-PA) and simultaneous reduction of plasminogen activator-inhibitor 1 (PAI-1) displays the driving force of hyperfibrinolysis, which finally concludes into a disseminated intravascular coagulopathy (DIC).

The current European guidelines on the treatment of major bleeding and coagulopathy following trauma recommend a target hemoglobin level of 70–90 g/L and an early initiation of coagulation management, the avoidance of hypothermia and acidosis, and the transfusion of blood products [1]. The early administration of fibrinogen, guided by point-of-care diagnostics, has gained wide acceptance and is therefore recommended for the treatment of major hemorrhage, whereas the substitution of coagulation factor XIII (FXIII) is only supported by a low level of evidence. However, two recent studies were able to show an increase in survival and a reduced need for transfusion by implementing a multimodal coagulation algorithm that included FXIII replacement in severe trauma patients [12,13]. Nonetheless, little in-vivo data investigating the effect of FXIII substitution on the coagulatory function of trauma patients are available. This is partly reasoned by the high demand for blood and coagulatory products, making it almost impossible to identify the isolated effect of FXIII substitution.

For this reason, thrombelastographic in-vitro models offer the opportunity to systematically investigate coagulatory interventions. Ågren et al. aimed to identify the optimal restoration of coagulatory function during major hemorrhage by investigating the effect of varying red blood cell concentrate (RBC)/fresh frozen plasma (FFP)/platelet concentrate ratios on dilutional coagulopathy [14,15]. This experimental setup was chosen in order to simulate massive transfusion. In contrast, other study setups analyzed native blood samples that were diluted with different types of colloids, and then investigated the treatment effect of FXIII on dilutional coagulopathy [16,17]. However, the use of blood samples of healthy donors instead of reconstituted blood makes them barely comparable to the clinical setting of major hemorrhage with the need for massive transfusion [17,18]. A further study investigated the effect of a 60% dilution on blood samples, which reflects an extreme hemodilution and is not commonly seen in trauma patients [16]. For this reason, this study aimed to combine the mentioned study setups in order to answer the question of whether combined fibrinogen/FXIII administration performs superiorly to sole fibrinogen therapy for the treatment of dilutional coagulopathy. First, massive transfusion is simulated by using reconstituted blood with subsequent hemodilution. Second, dilutional coagulopathy is treated with sole fibrinogen administration or combined fibrinogen/FXIII therapy.

## 2. Materials and Methods

### 2.1. Study Design and Recruitment of Study Probands

This study was approved by the local ethics committee (AZ 171/18) and performed in accordance with the Strengthening the Reporting of Observational Studies in Epidemiology (STROBE) criteria. Informed consent was signed prior to blood donation. Donors were asked for their medical history before blood withdrawal. In cases featuring a positive history for coagulatory diseases or any other severe chronic health condition, as well as the use of acetylsalicylic acid, other non-steroidal anti-inflammatory drugs, or any other kind of anticoagulants within the last seven days, the probands were excluded for this study.

### 2.2. Processing of Blood Products

The blood of ten healthy donors was recruited in the local Department of Transfusion Medicine in order to gain RBC concentrates. Blood was drawn of the antecubital vein and processed according to the standard operating procedure of the local Department of Transfusion Medicine. FFP samples and platelets were recruited from regular storage of the same department. Blood samples were processed according to German guidelines on the storage of blood products [19]. Samples of RBC were processed within three months after the collection of samples.

### 2.3. Experimental Setup

**Figure 1:**
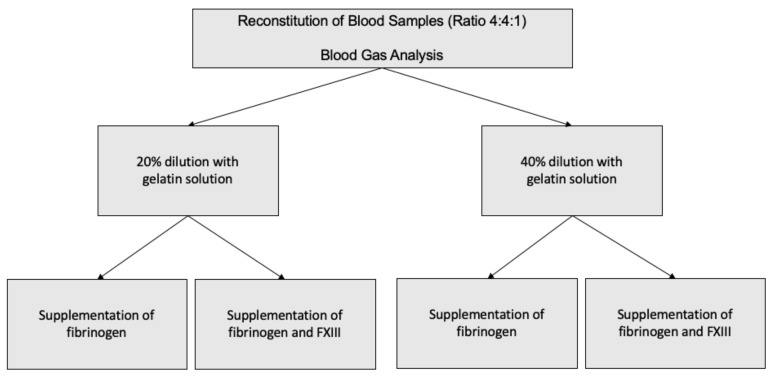
Visualization of the study setup.

The experimental study setup is presented in Figure 1. Two aliquots of 10 mL were drawn from RBC and platelet concentrates as well as of the FFP and reconstituted in each of five citrate (1.8 mL) and hirudin (1.6 mL) tubes (Sarstedt, Nuembrecht, Germany). For comparability to realistic transfusion protocols and to previous studies, we chose a 4:4:1 ratio for reconstitution (2 mL of RBC, 2 mL of FFP, 0.5 mL of platelet concentrate). Reconstituted blood consisted of FFP and RBC concentrates of the same blood group. In order to a achieve hemodilution of 20%, respectively, 40% of the reconstituted blood was replaced with a corresponding amount of gelatin 4% (Gelafundin^®^, B. Braun Melsungen AG, Melsungen, Germany). Immediately after dilution, samples were further processed with thrombelastometric and impedance aggregometric measurements. Further, each reconstituted blood sample was accompanied by a blood gas analysis for quality control.

After dilution of the reconstituted blood, samples were treated either with fibrinogen (Haemocomplettan^®^ 1 g (20 mg/mL), CSL Behring, Marburg, Germany) or a combination of fibrinogen and FXIII (Fibrogammin^®^ 250 International Units (IU) (62,5 IU/mL), CSL Behring, Marburg, Germany). The dosages were calculated corresponding to an adult with a bodyweight of 70 kg and an estimated blood volume of 4900 mL (70 mL/kg). Fibrinogen dosage was 50 mg/kg, leading to a total volume of 65 µL of fibrinogen for 1.8 mL citrate probes and 58 µL for 1.6 mL hirudin probes, respectively. FXIII administration was calculated corresponding to a dosage 75 IU/kg (32 µL in 1.8 mL citrate probes and 28.5 µL in 1.6 mL hirudin probes). In order to simulate massive transfusion-associated acidosis, we relinquished to correct pH-compensation.

### 2.4. Measurements

Thrombelastography (ROTEM^®^ delta, Matel Medizintechnik, Hausmannstätten, Germany), impedance aggregometry (Multiplate^®^ Analyzer, Roche, Basel, Switzerland), and blood gas analyses (Radiometer ABL800 FLEX, Radiometer, Krefeld, Germany) were performed by using point-of-care devices according to the individual manufacturers’ instructions. A quality check of all devices was performed according to the manufacturers’ instructions on a routine daily basis, and all used reagents were prepared immediately before processing.

Thrombelastographic reagents consisted of INTEM and EXTEM in order to obtain visibility of the coagulatory function of the intrinsic (INTEM) and extrinsic (EXTEM) pathways, while FIBTEM and APTEM were used to investigate fibrinogen-dependent coagulation (FIBTEM) and the extent of fibrinolysis (APTEM). At first, citrate samples were treated with the star-TEM reagent in order to recalcificate the blood and were then were treated with the mentioned reagents. In detail, by adding the EXTEM reagent, the extrinsic coagulation pathway was activated by the tissue factor, while phospholipid and ellagic acid of the INTEM reagent were used for the activation of the intrinsic coagulation pathway. Additionally, fibrinogen-dependent coagulation was stimulated with the cytochalasin D-containing FIBTEM reagent, while fibrinolysis was inhibited by the aprotinin-containing APTEM reagent [20]. Further, validated thrombelastographic values for the initiation of coagulation (clotting time (CT) in seconds, clot firmness time (CFT) in seconds), clot strength (mean clot firmness (MCF) in mm), and fibrinolysis (lysis index after 60 min (LI60) in %) were recorded [21].

Impedance aggregometry was used to describe platelet aggregation in whole blood samples. With the aid of an automatic pipette, either thrombin receptor activating peptide (TRAPtest, Verum Diagnostica GmbH, Munich, Germany), adenosine diphosphate (ADPtest, Verum Diagnostica GmbH, Munich, Germany), or arachidonic acid (ASPItest, Verum Diagnostica GmbH, Munich, Germany) was added according to the system’s instructions. In order to describe the aggregation capacity, the area under the curve of the impedance aggregometry (Units (U)) was recorded.

Blood gas analysis included the measurement of pH, levels of hemoglobin (g/dL), hematocrit (%), sodium (mmol/L), potassium (mmol/L), calcium (mmol/L), chloride (mmol/L), lactate (mmol/L), glucose (mg/dL), pO2 (mmHg), and pCO2 (mmHg).

### 2.5. Statistical Analysis

First, a descriptive evaluation of the blood gas analyses was performed in order to verify sufficient reconstitution and in the following efficiency of the dilutional steps. The Shapiro–Wilk test was used to determine the normal distribution of the measured values. In the case of normal distribution mean and standard deviation, median and interquartile range (IQR) for non-normally distributed values, respectively, were calculated. For multi-group analyses of non-normally distributed data, the Friedman test was used, followed by the Wilcoxon test for the determination of intergroup differences (in the case of positive Friedman testing), while normally distributed data were analyzed for this purpose by ANOVA and T-test. A value of *p* < 0.05 was considered to be statistically significant. All statistical analyses were performed using R statistical software version 4.0.2 (www.r-project.org, accessed on 4 September 2020).

## 3. Results

### 3.1. Characteristics of the Study Probands

Overall, one female and nine male healthy blood donors were included in the study. The median age of blood donors was 28 (24.5–32.3) years. The median activity of FXIII amounted to 87 (82–104%), which was within the local reference ranges for adults (FXIII activity, 75–150%).

### 3.2. Effects of Hemodilution

A significant reduction of hematocrit indicated effective dilution of the samples (native: 26.2 (25.9–28.8); 20% dilution: 6.8 (6.5–7.2); 40% dilution: 5.2 (4.5–6); both *p* < 0.01). Native reconstitution of the blood samples was associated with acidosis, which was aggravated by neither 20% nor 40% dilution of samples (pH native: 7.02 (7–7.06); 20% dilution: 7.04 (7.01–7.06); 40% dilution: 7.05 (7.02–7.06); both *p* = 0.26).

Native blood reconstitution showed physiological thrombaggregometric values (Table 1). While only minor changes of EXTEM and FIBTEM MCF were detectable after 20% dilution (EXTEM: *p* = 0.03; FIBTEM: *p* = 0.06; Table 1), after 40% dilution, INTEM, EXTEM, and FIBTEM MCF were significantly decreased (INTEM: *p* = 0.01; EXTEM: *p* = 0.01; FIBTEM: *p* < 0.01; Table 1). Compared to the native reconstitution, neither CT and CFT nor LI60 changed significantly after diluting the samples by 20%; whereas INTEM CFT, as well as FIBTEM and APTEM CT, significantly increased after 40% dilution (INTEM CFT: *p* < 0.01; FIBTEM CT: *p* = 0.05; APTEM CT: *p* = 0.02; Table 1).

Further, the reconstitution of blood samples led to a significant impairment of the aggregometry results. Interestingly, the dilution of blood samples was associated with a limited but still significant increase of all three aggregometric parameters (Supplementary Table S1).

### 3.3. Treatment of Dilutional Coagulopathy with Fibrinogen or Combination of Fibrinogen and FXIII

#### 3.3.1. Clot Properties

**Figure 2:**
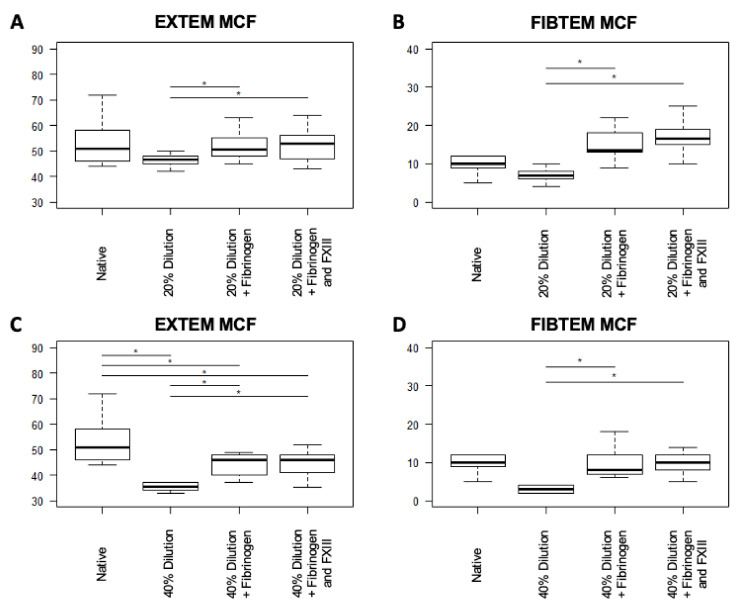
Boxplot diagram showing the results of the administration of fibrinogen and combined fibrinogen/FXIII to samples that were diluted by 20% (**A**,**B**) and 40% (**C**,**D**), respectively. Significant differences are highlighted with an asterisk (*: *p* < 0.05). Abbreviations: CT = clotting time; CFT = clot formation time; LI60 = lysis index after 60 min; MCF = mean clot firmness.

Within samples, which were diluted by 20%, FIBTEM MCF increased significantly and independently of the treatment arm (both *p* < 0.01; Table 2). With the exception of INTEM MCF after fibrinogen/FXIII-treatment, all tests showed minor changes of the MCF, which nonetheless reached statistical significance (for Fibrinogen, INTEM MCF: *p* = 0.04; EXTEM MCF: *p* = 0.02; APTEM MCF: *p* = 0.03; for Fibrinogen/FXIII, EXTEM MCF: *p* = 0.02; APTEM MCF: *p* = 0.01; Table 2). Compared to the isolated administration of fibrinogen, the combined treatment with fibrinogen/FXIII led to improved FIBTEM and EXTEM MCF, but failed to reach statistical significance, whereas APTEM MCF showed significant improvement after combined fibrinogen/FXIII-treatment in 20% dilution (FIBTEM: *p* = 0.1; EXTEM: *p* = 0.36; APTEM: *p* < 0.01; Table 2, Figure 2).

Overall, the FIBTEM MCF of the 40%-diluted blood samples increased significantly in both treatment groups (both *p* < 0.01; Table 3), but between both treatment arms, only minor changes of FIBTEM MCF were detectable (*p* = 0.1; Table 3). However, independently of the extent of dilution and the analyzed reagent, MCF did not differ between the fibrinogen and combined fibrinogen/FXIII therapy (Table 3, Figure 2).

#### 3.3.2. Clot Formation

Within blood samples of 20% dilution, both treatment arms were associated with shorter CT in EXTEM, FIBTEM, and APTEM reagents. Only the improvement of FIBTEM CT of the isolated fibrinogen treatment as well as the EXTEM CT after combined fibrinogen/FXIII treatment did not reach a level of statistical significance (for Fibrinogen, EXTEM CT: *p* = 0.03; FIBTEM CT: *p* = 0.65; APTEM CT: *p* = 0.02; for Fibrinogen/FXIII, EXTEM CT: *p* = 0.06; FIBTEM CT: *p* = 0.04; APTEM CT: *p* = 0.02; Table 2). Regarding the CFT, opposing results between the two treatment arms were identified. While isolated fibrinogen administration was associated with prolonged EXTEM and APTEM CFT, they were significantly reduced by the therapy with fibrinogen/FXIII (for Fibrinogen, EXTEM CFT: *p* = 0.04; APTEM CT: *p* = 0.02; APTEM CFT: *p* = 0.01; for Fibrinogen/FXIII, EXTEM CFT: *p* = 0.04; APTEM CT: *p* = 0.02; APTEM CFT: *p* < 0.01; Table 2).

The results of the blood samples that were diluted by 40% showed similar results. First, treatment with fibrinogen led to a slight reduction of INTEM CT and a distinct reduction of the CT of all other reagents (for Fibrinogen, INTEM CT: *p* = 0.02; EXTEM CT: *p* < 0.01; FIBTEM CT: *p* = 0.02; APTEM CT: *p* < 0.01; Table 3). On the other hand, combined fibrinogen/FXIII administration only reduced CT in the FIBTEM and APTEM measurements (for Fibrinogen/FXIII, INTEM CT: *p* = 0.06; EXTEM CT: *p* < 0.01; FIBTEM CT: *p* = 0.02; APTEM CT: *p* < 0.01; Table 3). Overall, CFT was prolonged, but only reached statistical significance in the INTEM (both treatment arms: INTEM CFT: *p* < 0.01; Table 3) and APTEM measurements (combined fibrinogen/FXIII: *p* = 0.04; Table 3). Most importantly, regarding CT and CFT, no differences were detectable between the two treatment arms.

#### 3.3.3. Lysis Capacity

Independently of 20% or 40% dilution, neither the EXTEM nor the APTEM LI60 was significantly reduced between the treatment arms (Table 2 and Table 3).

## 4. Discussion

This observational study was performed in order to identify the potential benefit of a combined fibrinogen/FXIII therapy for the treatment of dilutional coagulopathy by the use of an in-vitro massive transfusion model. In contrast to previous studies, this investigation was based on reconstituted blood in order to simulate massive transfusion rather than to dilute the blood of healthy donors [16,17,22,23,24,25]. The study was able to show the negative effect of hemodilution by identifying alterations of the thrombelastography, which were unsurprisingly more severe in the samples diluted by 40%. In particular, the INTEM and EXTEM MCF were significantly reduced, which is generally associated with reduced clot firmness deriving from a lack of fibrinogen, FXIII, and platelets [26,27,28]. Interestingly, the clotting time of all reagents was reduced after 20% dilution and only prolonged after 40% dilution, however, without reaching statistical significance. On the other hand, the CFT of all reagents was increased already after 20% dilution. These results are in line with previous reports and indicate that 20% dilution does not yet affect the initiation of coagulation (CT), but prolongs already the time to create a solid clot of 20 mm strength (CFT) [29,30,31]. Since the CFT, contrary to the CT, is highly dependent on sufficient platelet aggregation, these results indicate that hemodilution might have affected the concentration of platelets and their function [21,32]. The LI60 was not affected by the hemodilution, indicating that no hyperfibrinolysis was present in this study. Based on the presence of a dilutional coagulopathy, this study confirmed the therapeutic effect of fibrinogen on clot formation and stability. Several thrombelastographic parameters were optimized by the administration of fibrinogen, but most importantly, clinically relevant parameters also showed improvement. For example, FIBTEM and EXTEM MCF as indicators for the clot strength were significantly increased after fibrinogen substitution, but also the time to clot initiation (CT) as well as the time to clot generation (CFT) was improved in the EXTEM and APTEM measurements. While fibrinogen substitution comparable to 50 mg/kg in humans sufficiently normalized the mentioned parameters within the 20%-diluted blood samples, deficiencies were still identifiable in the 40%-diluted blood samples [26]. Since the clot formation (CFT) and clot strength (MCF) are not only dependent on fibrinogen but also on platelet aggregation, the reduced restoration of thrombelastographic parameters after 40% might be explainable with a hemodilution-induced alteration of platelet count and function. However, this study was not able to detect a significant difference between the isolated substitution of fibrinogen and the combined treatment with fibrinogen and FXIII, questioning the therapeutic benefit of FXIII for dilutional coagulopathy. It has to be emphasized that FXIII activity was within the normal range prior to the beginning of the experiments indicating that the lacking therapeutic effect of combined fibrinogen/FXIII supplementation was not caused by FXIII-deficiency.

The findings of this study are in contrast to the results of Schlimp et al., who investigated the influence of fibrinogen and FXIII in an equivalent in-vitro dilutional model [17]. Interestingly, they found only minor changes in clot stability after dilution with saline, while fibrin polymerization was severely disturbed after dilution with colloids. In contrast to our results, in blood samples that were diluted with gelatin, the combination of fibrinogen and FXIII led to stronger FIBTEM MCF compared to the sole substitution of fibrinogen. Further, Schlimp et al.’s findings were not reproducible in blood that had been diluted with hydroxyethyl starch. However, there were significant differences between both studies. First, Schlimp et al. diluted the blood of healthy donors by 33%, which does not reflect the situation in patients suffering from major hemorrhage because their resuscitation frequently involves massive transfusion protocols, with complete replacement of the circulating blood volume. In order to reflect this clinical setting, we chose to dilute reconstituted blood rather than to dilute the blood of healthy donors. Nevertheless, even though the used FFPs were processed rapidly according to the German transfusion guidelines, this study setup might have led to decreased levels of coagulation factors within the samples. Second, while fibrinogen dosages were comparable in both studies, the dosage of FXIII was almost twice as high as in our study (comparable to 142 vs. 75 IU/kg in humans). This has to be underlined because we already chose to use a dosage that is higher than the recommended dosage of 40 IU/kg for the perioperative substitution in patients suffering from FXIII deficiency [33]. Further, Innerhofer et al. demonstrated a potential benefit of a transfusion protocol including FXIII with a dosage of 20 IU/kg, leading to a median dosage of 27 IU/kg in the intervention group [13]. For this reason, we assumed that a dosage of 75 IU/kg should be sufficient for the treatment of FXIII deficiency in patients suffering from a major hemorrhage. The varying success rate of FXIII therapy might be explainable by the study of Nagashima et al., which showed that only ultra-high dosages of FXIII were able to improve clot firmness in an in-vitro hyperfibrinolysis model [23]. However, this study did not include the substitution of fibrinogen, which might be a reason why lower FXIII dosages did not result in improvement of MCF. Again, this study was performed with the blood of healthy volunteers and not with reconstituted blood. These findings were further supported by another study, in which only high dosages of fibrinogen and FXIII trended to an improved clot firmness [22]. Theusinger et al. investigated FXIII supplementation in samples deriving from intensive care patients with high fibrinogen but low FXIII levels and concluded that only supraphysiologic levels of FXIII led to an increase of FIBTEM MCF [34]. In contrast, a study by Kind et al. was able to improve clot stability in gelatin-induced dilutional coagulopathy by comparably low dosages of FXIII (1250 IU/70 kg). Yet, they again used the blood of healthy volunteers and reached FXIII levels > 60%, which might explain the improved clot firmness [16]. Also, Hanna et al. showed, with the help of an in-vitro dilutional model of healthy volunteers, that clinically established dosages of FXIII did not increase clot firmness, whereas FIBTEM MCF was improved by combined fibrinogen/FXIII supplementation [35]. The use of albumin for hemodilution might explain these opposing results, because albumin is known to impair coagulation to a higher degree than other colloids [15,36]. In summary, these findings support the hypothesis that during massive transfusion, next to fibrinogen, an ultra-high dosage of FXIII is needed for the effective therapy of dilutional coagulopathy [22,23,34].

Impedance aggregometry showed severe impairment of all measurements, which was already apparent in the native blood reconstitution, but also in both dilutions. Surprisingly, dilution of blood samples was associated with a minor improvement of platelet aggregation. Unfortunately, only limited data are available for the interpretation of these findings. To our knowledge, the only comparable study by Kind et al. also demonstrated a severely impaired platelet function after 60% dilution of blood samples, independently of the analyzed reagent [16]. In contrast to our study, Kind et al. analyzed the blood of healthy volunteers, which might explain superior platelet function in the undiluted blood samples. Even though the reconstitution of blood with a ratio of 4:4:1 (RBC:FFP:platelets) has been validated in previous studies, a reduced number of platelets might offer an explanation for the severe impairment of impedance aggregometry in our study [15]. While it is under discussion whether RBC and FFP concentrates should be transfused in a 1:1 or 2:1 ratio, the optimal ratio for the transfusion of platelets still remains unclear [1]. Further, gelatin might also reduce platelet function by modulating the glycoprotein IIb/IIIa receptor [37]. Last, acidosis, as present in our samples, can lead to significant impairment of platelet function [38,39]. As a consequence of the severe impairment of the impendence aggregometry, we chose not to include it in the further experiments involving fibrinogen and FXIII.

This study has some limitations, though. First, the transfusion regimen of 4:4:1 can only resemble the real-life clinical situation of massive transfusion. For this reason, the conclusions of this study have to be validated in prospective in-vivo studies. Second, this study provides information on neither the baseline nor the reached level of fibrinogen and FXIII in the reconstituted blood samples. While fibrinogen administration was effective in terms of normalization of the FIBTEM MCF, it remains unclear if the FXIII dosage led to sufficient FXIII levels. Third, hyperfibrinolysis, which represents a common problem during severe hemorrhage, was not simulated in this experimental model, indicated by regular results of APTEM analyses. This might indicate an important issue because FXIII exhibits close interactions to α2-antiplasmin and therefore offers antifibrinolytic effects [40]. The lack of hyperfibrinolysis might thus offer an explanation as to why FXIII supplementation had no beneficial effect on clot firmness. Fourth, since gender differences can affect the results of thrombelastometry, the study offers a bias caused by the predominantly male blood donors [41,42]. Especially, FIBTEM MCF is of high clinical interest during massive bleeding and displays a target parameter for fibrinogen substitution. Even though thrombelastographic parameters are known to vary between the genders, the difference in FIBTEM MCF is of minor extent. However, it has to be emphasized that the results might have been altered by gender bias. Beyond the gender-specific differences, the coagulatory function and, therefore, also the thrombelastography show typical age-related changes. For this reason, the results of this study are only transferable to the age group of young adults, while further studies should address older and younger age groups [41]. Last, acidosis is associated with coagulatory dysfunction and was present in our blood samples. This problem has already been described by other studies with similar study designs, and is reasoned by the low pH of RBC concentrates [15,43,44]. However, physiologic coagulatory function has been shown in the native blood reconstitution.

## 5. Conclusions

Compared to sole fibrinogen therapy, no beneficial effect of the combined administration of fibrinogen and FXIII for the treatment of dilutional coagulopathy was detected in this in-vitro model of massive transfusion. The result was independent of the degree of dilution. Previous studies suggest that ultra-high dosages of FXIII might be necessary for improved clot stability [22,23,34]. However, these studies used healthy blood samples, which are hardly representative of major hemorrhage. For this reason, ultra-high dosages of FXIII should be evaluated in in-vitro massive transfusion models.

## Figures and Tables

**Table 1:** Descriptive analysis of thrombelastography results.

Parameter	Native	Dilution 20%	Dilution 40%	*p*-Value Native vs. 20%	*p*-Value Native vs. 40%
INTEM CT (s)	220 (214–255)	196.5 (180–207.3)	250 (236–268.8)	0.11	0.57
INTEM CFT (s)	173 (147–186)	196.5 (153.3–235.8)	462.5 (414.3–593.3)	0.16	***<0.01***
INTEM MCF (mm)	52 (50–53)	45.5 (42.3–47.8)	30 (29–32.5)	0.12	***0.01***
INTEM LI60 (%)	99 (98–100)	97 (96–99)	95 (95–99)	0.71	0.28
EXTEM CT (s)	92 (67–102)	72.5 (66.3–94)	119 (88.3–125.8)	0.55	0.55
EXTEM CFT (s)	160 (119–242)	213.5 (203.3–255)	388 (367–434)	0.29	0.29
EXTEM MCF (mm)	51 (46–58)	46.5 (45.3–47.8)	35.5 (34.3–36.8)	***0.03***	***0.01***
EXTEM LI60 (%)	100 (97.5–100)	99 (96.8–100)	99 (96–100)	1.0	1.0
FIBTEM CT (s)	71 (59–86)	63.5 (58.5–87.5)	265 (130–570)	0.81	***0.05***
FIBTEM MCF (mm)	10 (9–12)	7 (6.3–7.8)	3 (2–3.8)	0.06	***<0.01***
FIBTEM LI60 (%)	100 (100)	100 (100)	100 (100)	n.c.	1.0
APTEM CT (s)	67 (63–83)	70.5 (64.5–79.3)	100 (89–145)	0.86	***0.02***
APTEM CFT (s)	175 (146–236)	226 (201.3–264.5)	413 (325–455)	0.57	0.29
APTEM MCF (mm)	49 (46–52)	46 (42.3–47.8)	40 (35–40)	0.48	0.22
APTEM LI60 (%)	100 (100)	99 (97.3–100)	100 (97–100)	0.56	1.0

Shown *p*-values represent either the differences between the native reconstituted blood and the 20% dilution and 40% dilution, respectively. Significant differences are highlighted in italics and bold. Abbreviations: CT = clotting time; CFT = clot formation time; LI60 = lysis index after 60 min; MCF = mean clot firmness; n.c. = not calculable).

**Table 2:** Treatment of blood samples that were diluted by 20% and treated either with fibrinogen or combined fibrinogen/FXIII.

Parameter	Dilution 20%	Dilution 20% Fibrinogen	Dilution 20% Fibrinogen + FXIII	*p*-Value Dilution 20% vs. Fibrinogen	*p*-Value Dilution 20% vs. Fibrinogen + FXIII	*p*-Value Dilution 20% Fibrinogen vs. Fibrinogen + FXIII
INTEM CT (s)	196.5 (180–207.3)	224 (211.5–267.5)	213.5 (203–246.5)	0.19	0.19	0.49
INTEM CFT (s)	196.5 (153.3–235.8)	155 (128.5–182.5)	140 (114.5–194.8)	0.08	0.16	0.77
INTEM MCF (mm)	45.5 (42.3–47.8)	49.5 (46.3–53.8)	51 (44.8–54.8)	***0.04***	0.12	*0.34*
INTEM LI60 (%)	97 (96–99)	99 (98–99)	100 (99.8–100)	0.30	0.30	0.37
EXTEM CT (s)	72.5 (66.3–94)	54 (50.3–57.3)	51 (45.5–53.5)	***0.03***	0.06	0.36
EXTEM CFT (s)	213.5 (203.3–255)	175 (136.5–217.3)	141.5 (117.3–192.5)	***0.04***	***0.04***	0.11
EXTEM MCF (mm)	46.5 (45.3–47.8)	50.5 (48.3–54.8)	53 (48.3–55.8)	***0.02***	***0.02***	0.36
EXTEM LI60 (%)	99 (96.8–100)	100 (100)	100 (100)	0.09	0.09	n.c.
FIBTEM CT (s)	63.5 (58.5–87.5)	48.5 (46.5–50)	50.5 (43–57)	0.65	***0.04***	0.84
FIBTEM MCF (mm)	7 (6.3–7.8)	13.5 (13–17.3)	16.5 (15.3–18.8)	***<0.01***	***<0.01***	*0.1*
FIBTEM LI60 (%)	100 (100)	100 (100)	100 (100)	n.c.	n.c.	n.c.
APTEM CT (s)	70.5 (64.5–79.3)	53 (51–59)	49 (48.3–52.8)	***0.02***	***0.02***	0.81
APTEM CFT (s)	226 (201.3–264.5)	191 (119–219)	132.5 (93–176)	***0.01***	***<0.01***	***0.01***
APTEM MCF (mm)	46 (42.3–47.8)	50 (49–55)	56.5 (51.8–60.8)	***0.03***	***0.01***	***0.01***
APTEM LI60 (%)	99 (97.3–100)	100 (99.5–100)	100 (100)	0.09	0.09	1.0

Significant differences are highlighted in italics and bold. Abbreviations: CT = Clotting Time; CFT = Clot Formation Time; LI60 = lysis index after 60 min; MCF = mean clot firmness; n.c. = not calculable.

**Table 3:** Treatment of blood samples which were diluted by 40% and treated either with fibrinogen or combined fibrinogen/FXIII.

Parameter	Dilution 40%	Dilution 40% Fibrinogen	Dilution 40% Fibrinogen + FXIII	*p*-Value Dilution 40% vs. Fibrinogen	*p*-Value Dilution 40% vs. Fibrinogen + FXIII	*p*-Value Dilution 40% Fibrinogen vs. Fibrinogen + FXIII
INTEM CT (s)	250 (236–268.8)	218 (206.3–234)	231.5 (208.3–256))	***0.02***	0.06	0.84
INTEM CFT (s)	462.5 (414.3–593)	265 (212.8–354)	259 (213–400)	***<0.01***	***<0.01***	0.82
INTEM MCF (mm)	30 (29–32.5)	42 (38–45.8)	41.5 (36–44.5)	***0.02***	0.18	1.0
INTEM LI60 (%)	95 (95–99)	100 (98.8–100)	100 (99.8–100)	0.16	0.16	1.0
EXTEM CT (s)	119 (88.3–125.8)	52 (51–59.8)	51.5 (51–53.8)	***<0.01***	***<0.01***	0.29
EXTEM CFT (s)	388 (367–434)	286.5 (246.5–403.3)	286.5 (228.3–355.5)	0.11	0.08	0.31
EXTEM MCF (mm)	35.5 (34.3–36.8)	46 (40.3–47.8)	46 (41.5–48)	***<0.01***	***<0.01***	0.21
EXTEM LI60 (%)	99 (96–100)	100 (100)	100 (100)	0.41	0.29	1.0
FIBTEM CT (s)	265 (130–570)	53 (52–57.3)	54 (46.3–60)	***0.02***	***0.02***	0.76
FIBTEM MCF (mm)	3 (2–3.8)	8 (7–11.3)	10 (8.3–11.8)	***<0.01***	***<0.01***	*0.78*
FIBTEM LI60 (%)	100 (100)	100 (100)	99 (83.5–100)	n.c.	0.36	0.2
APTEM CT (s)	100 (89–145)	55 (51–59.8)	53 (50.3–56.8)	***<0.01***	***<0.01***	0.88
APTEM CFT (s)	413 (325–455)	300.5 (262.8–455.8)	313 (228.8–381)	0.07	***0.04***	0.07
APTEM MCF (mm)	40 (35–40)	45 (40.3–48.3)	45 (42–48.8)	***0.02***	***0.02***	0.38
APTEM LI60 (%)	100 (97–100)	100 (100)	100 (100)	0.37	0.37	n.c.

Significant differences are highlighted in italics and bold. Abbreviations: CT = Clotting Time; CFT = Clot Formation Time; LI60 = lysis index after 60 min; MCF = M=mean clot firmness; n.c. = not calculable.

## Data Availability

The data presented in this study are available on request from the corresponding author.

## Supplementary Materials

The following are available online at https://www.mdpi.com/article/10.3390/jcm10102068/s1, Table S1: Dilutional effects on the results of the impedance aggregometry.
