# Supplementary material for: Combined Administration of Fibrinogen and Factor XIII Concentrate Does Not Improve Dilutional Coagulopathy Superiorly Than Sole Fibrinogen Therapy: Results of an In-Vitro Thrombelastographic Study
Source: J Clin Med. 2021 May 12;10(10):2068. doi: 10.3390/jcm10102068 (PMC8150940; doi:10.3390/jcm10102068)
Supplement: Supplementary file 1 [file jcm-10-02068-s001.zip › jcm-1191972-supplementary.pdf]

Supplement

Table S1 Dilutional effects on the results of the impedance aggregometry. Parameters are presented as median (interquartile range). *Abbreviations: U = Units.*

**Parameter**	**Native**	**Dilution 20%**	**Dilution 40%**	***p*-value native vs. 20%**	***p*-value native vs. 40%**
ASPItest (U)	3 [3–4]	10 [9–14]	9 [7–10]	$p = 0.04$	$p = 0.04$
ADPtest (U)	2 [1–3]	11 [9–13]	9 [5–10]	$p = 0.04$	$p = 0.05$
TRAPtest (U)	6 [4–8]	15 [14–18]	14 [11–15]	$p = 0.04$	$p = 0.04$
